# Trifluoroethoxy-Coated Phthalocyanine Catalyzes Perfluoroalkylation of Alkenes under Visible-Light Irradiation †

**DOI:** 10.3390/molecules22071130

**Published:** 2017-07-07

**Authors:** Kohei Matsuzaki, Tomoya Hiromura, Hideki Amii, Norio Shibata

**Affiliations:** 1 Department of Nanopharmaceutical Sciences, Nagoya Institute of Technology Gokiso, Showa-ku, Nagoya 466-8555, Japan; cht11134@nitech.jp; 2 Department of Life Science and Applied Chemistry, Nagoya Institute of Technology Gokiso, Showa-ku, Nagoya 466-8555, Japan; 28411141@stn.nitech.ac.jp; 3 Division of Molecular Science, Graduate School of Science and Technology, Gunma University, 1-5-1 Tenjin-cho, Kiryu, Gunma 376-8515, Japan; amii@gunma-u.ac.jp

**Keywords:** phthalocyanine, photocatalysts, trifluoromethylation, perfluoroalkylation, visible light

## Abstract

We disclose herein the perfluoroalkylation of alkenes catalyzed by trifluoroethoxy-coated zinc phthalocyanine under irradiation of visible light. Perfluoroalkyl iodides were nicely incorporated into unsaturated substrates, including alkyne, to provide perfluoroalkyl and iodide adducts in moderate to good yields. Trifluoromethylation is also possible by trifluoromethyl iodide under the same reaction conditions. The mechanistic study is discussed.

## 1. Introduction

Perfluoroalkyl groups frequently appeared in the libraries of pharmaceuticals, agrochemicals, and functional materials and in the methods for the introduction of perfluoroalkyl groups to organic molecules, causing a massive accumulation of literature over the past few decades [1,2,3,4,5]. Radical perfluoroalkylation of alkenes using perfluoroalkyl halides (Rf-X) under shortwave UV irradiation is one of the classical and well-explored methods for this purpose [6,7,8]. However, the classical UV irradiation method [9,10,11,12] has often suffered from a lack of selectivity, low yields, and complicated reaction devices such as the quartz vessel or the merry-go-round reactor. In recent years, radical perfluoroalkylation has dramatically changed for the sake of discovery of photoredox catalyst systems under visible right irradiation [13,14,15,16,17,18,19,20,21,22,23,24,25,26,27,28,29,30,31]. The methods do not require complex reaction devices or harmful UV irradiation because environmentally benign visible lights and photocatalysts are used instead. Besides, high yields and high chemoselectivities are often observed under photo-catalysis without any harsh reaction conditions. Photoredox catalysts containing ruthenium or iridium complexed with polybipyridyl ligands absorbing blue light (λ = 375–450 nm) are mainly explored in this system [13,14,15,16,17,18,19,20,21,22,23,24,25,26,27]. In recent years, organic dyes such as eosinY or methylene blue have also started to be investigated as organic photoredox catalysts under blue to green light irradiation (λ = ca. 450–550 nm) [29,30,31]. Although several metal and non-metal photoredox catalysts have been developed, ruthenium or iridium complexes coordinated by polybipyridyl ligands are surely the most effective catalysts in these transformations, despite the major disadvantage of their high cost.

Phthalocyanines, which are man-made blue color dyes with nearly a century of history [32,33], are 18 π-electron macro-heterocycles consisting of four isoindoline units with a planar structure. Their large conjugated system induces good absorption bands of spectra at 620–700 nm, and their chemical, thermal, and photo stabilities, low-cost and non-toxicity makes them promising photosensitizers for dye-sensitized solar cell (DSSC) applications [34,35,36]. From the viewpoint of the successful application of phthalocyanines for DSSC, they should also be very attractive alternative catalysts to Ru(II) polypyridyl complexes for photoredox perfluoroalkylation reactions. In spite of their potential performance as photoredox materials, as mentioned above, research on phthalocyanines for photoredox radical perfluoroalkylation is rarely reported [37,38]. This is presumably due to the notorious low solubility of phthalocyanines in organic solvents [32,33]. In the last several years, we have reported the design and synthesis of a series of trifluoroethoxy-coated phthalocyanines, and revealed their extraordinary non-aggregation property allowing them to become highly soluble in a wide variety of organic solvents [39,40,41,42,43,44,45]. We recently reported that trifluoroethoxy-coated boron subphthalocyanine is a very effective catalyst for the radical fluoroalkylation of alkenes and alkynes under energetically lower red light irradiation [46]. However, apart from the advantages of its reactivity following red-light activation (λ = 600–700 nm), boron subphthalocyanine might have a problem, its long-term photo-stability [47,48,49]. That is, if the reaction requires very long time, catalytic activity would disappear. We disclose herein the radical perfluoroalkylation of alkenes, including alkyne, catalyzed by trifluoroethoxy-coated zinc phthalocyanine under visible light irradiation.

## 2. Results and Discussion

Initially, perfluorooctylation of 1-hexenol (**1a**) with perfluorooctyl iodide (*n*C_8_F_17_I) in the presence of a catalytic amount of trifluoroethoxy-coated zinc phthalocyanine (TFEO-ZnPc, 1 mol %) under LED light (white LED, 10 W) irradiation was attempted. The solvent system and additive were selected according to our previous report [46]. The desired perfluorooctylated product **2aa** was obtained after 1 h in 88% yield (Table 1, Entry 1). Control experiments showed the reaction no longer proceeded without light irradiation, catalyst, or additive (Entries 2–4). The uses of *t*Bu-functionalized zinc phthalocyanine (*t*BuZnPc) or trifluoroethoxy-coated subphthalocyanine (TFEO-SubPc) instead of TFEO-ZnPc decreased product yields (Entries 5, 6). Next, additives were screened and the use of ascorbic acid or Hantzsch ester resulted in a decrease in yields (Entries 7, 8). Finally, study of solvent effect revealed that single solvents such as MeOH, MeCN, or DMSO showed no improvement in yields (Entries 9–11), but an increase in concentration gave higher product yield (Entry 12).

**Figure 1:**
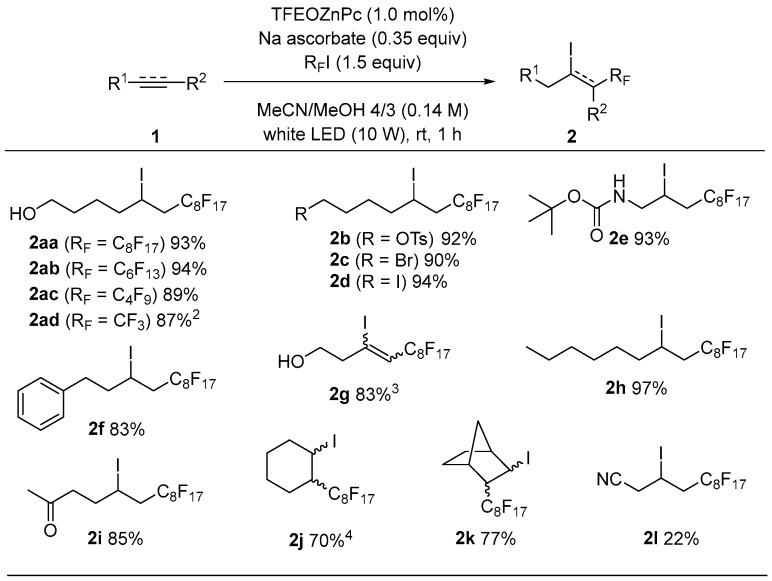
Perfluoroalkylation reaction of **1** with TFEO-ZnPc under visible light irradiation. The reaction of **1** (0.25 mmol) with perfluoroalkyliodide (0.375 mmol) was carried out in the presence of TFEO-ZnPc (0.0025 mmol) and Na ascorbate (0.0875 mmol) in MeCN (1.0 mL) and MeOH (0.75 mL) at room temperature under irradiation with white LED (10 W). Yields are shown as isolated yield. **2ad**: The reaction was carried out for 5 h with an excess amount of CF_3_I. **2g**: 3.7:1 dr. **2j**: 1.8:1 dr. R_F_I: perfluoroalkyliodide.

With optimized reaction conditions in hand, perfluoroalkylation of a variety of alkenes **1** in the presence of a catalytic amount of TFEO-ZnPc under visible light irradiation was attempted (Figure 1). Varied functionalized alkenes (**1**) having tosylate, halogens, carbamate, and ketone showed good reactivity to furnish perfluoroalkylated compounds (**2**) after 1 h irradiation. The reaction could be applied to inner-alkene substrates **1j** and **1k**, including alkyne **1g**, in comparable yields, and to electron-deficient alkene **1l** in acceptable yield. Other perfluoroalkyl iodides, including C4 and C6 perfluoroalkyl chains, were successfully used under the optimized reaction conditions and desired products **2ab** and **2ac** were afforded in 1 h. The trifluoromethylation reaction using trifluoromethyl iodide required a longer reaction time to furnish comparable product **2ad** in 87% yield.

**Figure 2:**
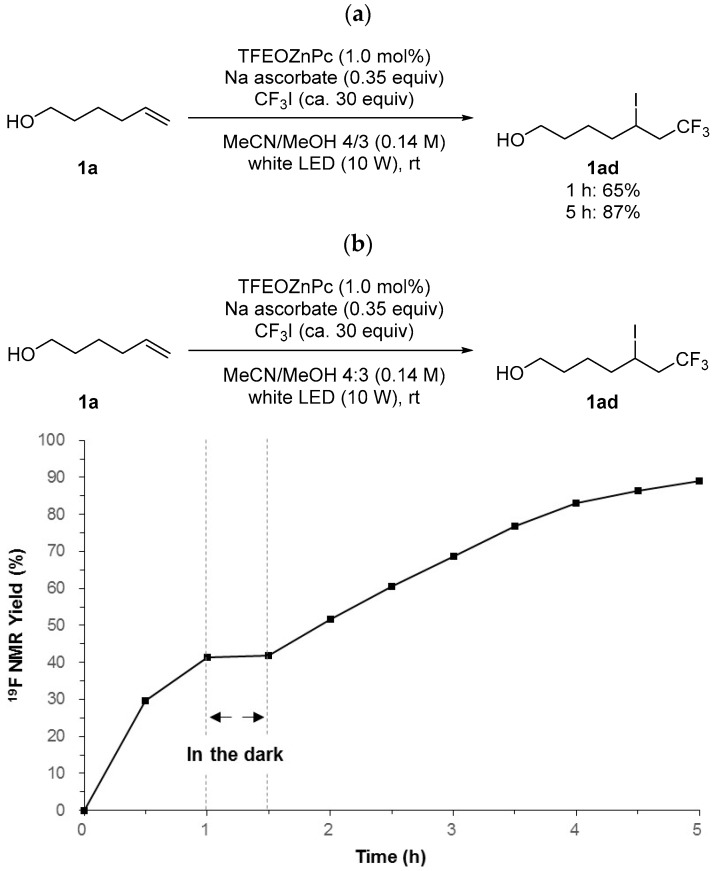
The time profile and light/dark experiment on trifluoromethylation of **1a** with TFEO-ZnPc under visible light irradiation: (**a**) Trifluoromethylation of **1a** with TFEO-ZnPc under optimized conditions for a reaction time of 1 h and 5 h; (**b**) Time profile and light/dark experiment on trifluoromethylation of **1a** with TFEO-ZnPc.

To confirm the reaction mechanism, the time profile of the reaction was investigated. The trifluoromethylation was selected for this purpose due to its longer reaction time (Figure 2). First, trifluoromethylation of **1a** was carried out with optimized conditions for only 1 h and 65% isolated yield of product **2ad** was obtained, even though an excess amount of CF_3_I was used (Figure 2a). This result indicates the difficulty of trifluoromethylation compared with other perfluoroalkylations. Next, the time profile was further studied by checking the yields of each reaction time with PhCF_3_ as an internal standard with a pause in light irradiation (Figure 2b). The reaction gradually proceeded and gave comparable yields after a 5 h reaction time, while the reaction did not proceed in the dark. These results show good agreement with our previous results [46] and with other reports [19] on the photo-induced radical trifluoromethylation of alkenes with photoredox catalysts.

**Scheme 1:**
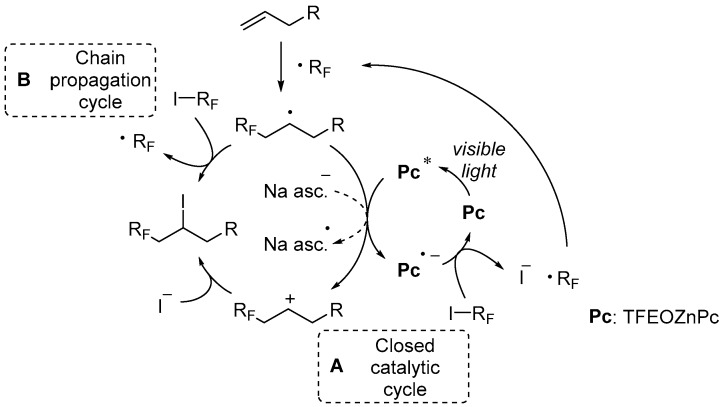
Plausible reaction mechanism of trifluoromethylation of alkenes with TFEO-ZnPc.

A plausible reaction mechanism shown in Scheme 1 is supported by previous reports [9] and by the light/dark experiment mentioned above. The reaction starts with the electron transfer from Na ascorbate to excited TFEO-ZnPc (Pc*) by visible light to form the TFEO-ZnPc anion radical (Pc^−^) and the anion radical reduces the perfluoroalkyliodide (R_F_I) to produce the perfluoroalkyl radical (R_F_). The radical reacts with an unsaturated moiety of the substrate to form an alkyl radical intermediate. Then, the alkyl radical may donate the electron to excited TFEO-ZnPc to reproduce the TFEO-ZnPc anion radical (Path A; Closed reaction cycle). Another possibility of this reaction is radical propagation of the perfluoroalkyl radical intermediate with R_F_I (Path B; Chain propagation cycle). The control experiment shows that both plausible reaction passes need an initial electron-transfer between Na ascorbate and TFEO-ZnPc and the experiment in Figure 1b shows that continuous light irradiation is essential for the production of a perfluoroalkylated product. From the previous study [46] and these results in this reaction, Path A and B may work concertedly in this transformation. Further studies are required to disclose the details of this mechanism.

## 3. Materials and Methods

All reactions were performed in oven-dried glassware under the positive pressure of argon unless otherwise mentioned. Solvents were transferred via syringe and were introduced into the reaction vessels though a rubber septum. All reactions were monitored by thin-layer chromatography (TLC) carried out on a 0.25 mm Merck silica gel (60-F_254_). TLC plates were visualized with UV light and KMnO_4_ in water/heat. Column chromatography was carried out on columns packed with silica gel (60N spherical neutral size 63–210 μm, Kanto Chemical Co., Inc., Tokyo, Japan). The ^1^H-NMR (300 MHz), ^19^F-NMR (282 MHz), and ^13^C-NMR (125 MHz) spectra for solution in CDCl_3_ were recorded on a Varian 300 (Agilent Technologies, Palo Alto, CA, USA) and a Bruker Avance 500 (Bruker, Billerica, MA, USA). Chemical shifts (δ) are expressed in ppm downfield from TMS (δ = 0.00) or C_6_F_6_ (δ = −162.2 (CDCl_3_)) as an internal standard. Mass spectra were recorded on a Shimadzu GCMS-QP5050A (EI-MS) and Shimadzu LCMS-2020 (ESI-MS) (Shimadzu Corporation, Kyoto, Japan). Melting points were recorded on a Buchi M-565 (Büchi Labortechnik AG, Flawil, Switzerland). Infrared spectra were recorded on a JASCO FT/IR-4100 spectrometer (Jasco Corporation, Tokyo, Japan). Chemicals were purchased and used without further purification unless otherwise noted. MeOH was dried and distilled before use.

All reactions were performed under irradiation by commercially available 10 W white LED (Panasonic Corporation, Osaka, Japan, DA10DGK60W, 810 lumens). The LEDs were placed at a distance of 3–4 cm.

### 3.1. Perfluoroalkylation of Alkenes and Alkynes with TFEOZnPc

A Schlenk tube equipped with a rubber septum and magnetic stir bar was charged with TFEO-ZnPc (5.4 mg, 0.0025 mmol, 1 mol %) and Na ascorbate (17.3 mg, 0.0875 mmol, 0.35 equiv). The tube was degassed by vacuum evacuation and argon backfill (×3) before MeCN (1.0 mL), MeOH (0.75 mL), substrate (0.25 mmol, 1.0 equiv) and perfluoroalkyliodide (0.375 mmol, 1.5 equiv) were added. The mixture was degassed by the freeze-pump-thaw method (×3). The mixture was stirred for 1 h under irradiation by 10 W white LEDs. After the reaction was complete, the mixture was diluted by Et_2_O and filtered through a pad of silica gel, and the filtrate was concentrated under reduced pressure. The crude product was purified by column chromatography on silica gel to give the desired product.

#### 3.1.1. 5-Iodo-6-perfluorooctylhexane-1-ol (**2aa**)

Following a general procedure, TFEO-ZnPc (5.4 mg, 0.0025 mmol, 1 mol %), Na ascorbate (17.3 mg, 0.0875 mmol, 0.35 equiv) alkene **1a** (29.5 μL, 0.25 mmol, 1.0 equiv) and C_8_F_17_I (99.0 μL, 0.375 mmol, 1.5 equiv) were used in MeCN (1.0 mL) and MeOH (0.75 mL) at room temperature for 1 h. The crude product was purified by column chromatography on silica gel (hexane/EtOAc = 8:2) to give perfluoroalkylated product **2a** (150.2 mg, 93% yield) as a white solid.

The ^1^H-NMR, ^19^F-NMR spectrum matched that reported in [19].

MS (EI, *m*/*z*) 519 [(M − I)^+^]; ^1^H-NMR (CDCl_3_, 300 MHz): δ 4.40–4.30 (m, 1H), 3.70–3.66 (m, 2H), 3.00–2.70 (m, 2H), 1.90–1.50 (m, 7H); ^19^F-NMR (CDCl_3_, 282 MHz): δ −81.2 (t, *J* = 9.0 Hz, 3F), −111.5–−112.5 (m, 1F), −114.5–−115.5 (m, 1F), −121.9 (br s, 2F), −122.3 (br s, 4F), −123.1 (br s, 2F), −123.9 (br s, 2F), −126.5 (br s, 2F).

#### 3.1.2. 5-Iodo-6-perfluorohexylhexanol (**2ab**)

Following a general procedure, TFEO-ZnPc (5.4 mg, 0.0025 mmol, 1 mol %), Na ascorbate (17.3 mg, 0.0875 mmol, 0.35 equiv) alkene **1a** (29.5 μL, 0.25 mmol, 1.0 equiv) and C_6_F_13_I (81.2 μL, 0.375 mmol, 1.5 equiv) were used in MeCN (1.0 mL) and MeOH (0.75 mL) at room temperature for 1 h. The crude product was purified by column chromatography on silica gel (hexane/EtOAc = 8:2) to give perfluoroalkylated product **2ab** (128.4 mg, 94% yield) as a white solid.

The ^1^H-NMR, ^19^F-NMR spectrum matched that reported in [19].

MS (EI, *m*/*z*) 419 [(M − I)^+^]; ^1^H-NMR (CDCl_3_, 300 MHz): δ 4.39–4.30 (m, 1H), 3.70–3.67 (m, 2H), 3.04–2.69 (m, 2H), 1.90–1.49 (m, 7H); ^19^F-NMR (CDCl_3_, 282 MHz): δ −81.3 (t, *J* = 9.9 Hz, 3F), −111.7–−112.7 (m, 1F), −114.7–−115.7 (m, 1F), −122.3 (br s, 2F), −123.4 (br s, 2F), −124.1 (br s, 2F), −126.7 (br s, 2F).

#### 3.1.3. 5-Iodo-6-perfluorobutylhexanol (**2ac**)

Following a general procedure, TFEO-ZnPc (5.4 mg, 0.0025 mmol, 1 mol %), Na ascorbate (17.3 mg, 0.0875 mmol, 0.35 equiv) alkene **1a** (29.5 μL, 0.25 mmol, 1.0 equiv) and C_4_F_9_I (63.0 μL, 0.375 mmol, 1.5 equiv) were used in MeCN (1.0 mL) and MeOH (0.75 mL) at room temperature for 1 h. The crude product was purified by column chromatography on silica gel (hexane/EtOAc = 8:2) to give perfluoroalkylated product **2ac** (99.3 mg, 89% yield) as a white solid.

The ^1^H-NMR, ^19^F-NMR spectrum matched that reported in [50].

MS (EI, *m*/*z*) 319 [(M − I)^+^]; ^1^H-NMR (CDCl_3_, 300 MHz): δ 4.39–4.30 (m, 1H), 3.71–3.67 (m, 2H), 2.98–2.69 (m, 2H), 1.89–1.48 (m, 7H); ^19^F-NMR (CDCl_3_, 282 MHz): δ −81.5 (t, *J* = 9.9 Hz, 3F), −111.9–−112.9 (m, 1F), −115.1–−116.0 (m, 1F), −125.1 (br s, 2F), −126.4 (br s, 2F).

#### 3.1.4. 5-Iodo-6-trifluoromethylhexanol (**2ad**)

A Schlenk tube equipped with a rubber septum and a magnetic stir bar was charged with TFEO-ZnPc (5.4 mg, 0.0025 mmol, 1 mol %) and Na ascorbate (17.3 mg, 0.0875 mmol, 0.35 equiv). The tube was degassed by vacuum evacuation and argon backfill (×3) before MeCN (1.0 mL), MeOH (0.75 mL) and alkene **1a** (29.5 μL, 0.25 mmol, 1.0 equiv) were added. The mixture was degassed by the freeze-pump-thaw method (×3). CF_3_I (1.45 g, 7.32 mmol, 29.3 equiv) in a balloon was then added to the tube via a needle then cooled to −78 °C in an ethanol bath. The mixture was warmed to room temperature and stirred for 5 h under irradiation by 10 W white LEDs. After the reaction was complete, the mixture was diluted by Et_2_O and filtered through a pad of silica gel, and the filtrate was concentrated under reduced pressure. The crude product was purified by column chromatography on silica gel (hexane/EtOAc = 8:2) to give desired product **2ad** (64.2 mg, 87% yield) as a white solid.

The ^1^H-NMR, ^19^F-NMR spectrum matched that reported in [51].

MS (EI, *m*/*z*) 169 [(M − I)^+^]; ^1^H-NMR (CDCl_3_, 300 MHz): δ = 4.25–4.16 (m, 1H), 3.71–3.66 (m, 2H), 2.98–2.74 (m, 2H), 1.86–1.44 (m, 7H); ^19^F-NMR (CDCl_3_, 282 MHz): δ = −64.4 (t, *J* = 10.4 Hz, 3F).

#### 3.1.5. 5-Iodo-6-perfluorooctylhexyll-4-methylbenzenesulfonate (**2b**)

Following a general procedure, TFEO-ZnPc (5.4 mg, 0.0025 mmol, 1 mol %), Na ascorbate (17.3 mg, 0.0875 mmol, 0.35 equiv) alkene **1b** (63.6 mg, 0.25 mmol, 1.0 equiv) and C_8_F_17_I (99.0 μL, 0.375 mmol, 1.5 equiv) were used in MeCN (1.0 mL) and MeOH (0.75 mL) at room temperature for 1 h. The crude product was purified by column chromatography on silica gel (hexane/EtOAc = 8:2) to give perfluoroalkylated product **2b** (184.3 mg, 92% yield) as a white solid.

m.p. = 54.3–55.3 °C; HRMS (EI) calcd. for C_21_H_18_F_17_O_3_S [(M − I)^+^]: 673.0705 found 673.0724; ^1^H-NMR (300 MHz, CDCl_3_): δ 7.82 (d, *J* = 8.3 Hz, 2H), 7.36 (d, *J* = 8.3 Hz, 2H), 4.29–4.21 (m, 1H), 4.05 (t, *J* = 6.2 Hz, 2H), 2.92–2.66 (m, 2H), 2.45 (s, 3H), 1.73–1.46 (m, 6H).; ^19^F-NMR (CDCl_3_, 282 MHz): δ −81.2 (t, *J* = 9.4 Hz, 3F), −111.6–−112.6 (m, 1F), −114.8–−115.8 (m, 1F), −122.1 (br s, 2F), −122.4 (br s, 4F), −123.3 (br s, 2F), −124.1 (br s, 2F), −126.6 (br s, 2F).; ^13^C NMR (CDCl_3_, 125 MHz): δ = 144.8, 133.0, 130.0, 127.9, 120.0–108.8 (m, C_8_F_17_), 69.9, 41.6 (t, *J* = 20.7 Hz), 39.4 (apparent doublet, *J* = 1.3 Hz), 27.8, 25.8, 21.6, 19.7; IR (KBr) 2940, 2362, 1599, 1352, 1202, 957, 812, 660, 557 cm^−1^.

#### 3.1.6. 1-Bromo-5-iodo-6-perfluorooctylhexane (**2c**)

Following a general procedure, TFEO-ZnPc (5.4 mg, 0.0025 mmol, 1 mol %), Na ascorbate (17.3 mg, 0.0875 mmol, 0.35 equiv), alkene **1c** (40.8 mg, 0.25 mmol, 1.0 equiv) and C_8_F_17_I (99.0 μL, 0.375 mmol, 1.5 equiv) were used in MeCN (1.0 mL) and MeOH (0.75 mL) at room temperature for 1 h. The crude product was purified by column chromatography on silica gel (hexane) to give perfluoroalkylated product **2c** (160.3 mg, 90% yield) as yellow oil.

The ^1^H-NMR, ^19^F-NMR spectrum matched that reported in [19].

HRMS (EI) calcd. for C_14_H_11_BrF_17_ [(M − I)^+^]: 580.9773 found 580.9785; ^1^H-NMR (CDCl_3_, 300 MHz): δ 4.37–4.28 (m, 1H), 3.42 (t, *J* = 6.6 Hz, 2H), 3.04–2.71 (m, 2H), 2.00–1.54 (m, 6H); ^19^F-NMR (CDCl_3_, 282 MHz): δ −81.1 (t, *J* = 9.9 Hz, 3F), −111.2–−112.3 (m, 1F), −114.2–−115.2 (m, 1F), −121.7 (br s, 2F), −122.0 (br s, 4F), −122.9 (br s, 2F), −123.7 (br s, 2F), −126.3 (br s, 2F).

#### 3.1.7. 1,5-Diiodo-6-perfluorooctylhexane (**2d**)

Following a general procedure, TFEO-ZnPc (5.4 mg, 0.0025 mmol, 1 mol %), Na ascorbate (17.3 mg, 0.0875 mmol, 0.35 equiv), alkene **1d** (52.5 mg, 0.25 mmol, 1.0 equiv) and C_8_F_17_I (99.0 μL, 0.375 mmol, 1.5 equiv) were used in MeCN (1.0 mL) and MeOH (0.75 mL) at room temperature for 1 h. The crude product was purified by column chromatography on silica gel (hexane) to give perfluoroalkylated product **2d** (178.3 mg, 94% yield) as yellow oil.

The ^1^H-NMR, ^19^F-NMR spectrum matched that reported in [19].

HRMS (EI) calcd. for C_14_H_11_F_17_I_2_ (M)^+^: 755.8679 found 755.8651; ^1^H-NMR (CDCl_3_, 300 MHz): δ 4.37–4.29 (m, 1H), 3.21–3.19 (m, 2H), 3.02–2.71 (m, 2H), 1.84–1.56 (m, 6H); ^19^F-NMR (CDCl_3_, 282 MHz): δ −81.2 (t, *J* = 9.3 Hz, 3F), −111.4–−112.3 (m, 1F), −114.5–−115.5 (m, 1F), −121.9 (br s, 2F), −122.3 (br s, 4F), −123.1 (br s, 2F), −123.9 (br s, 2F), −126.5 (br s, 2F).

#### 3.1.8. *tert*-Butyl (2-iodo-3-perfluorooctylpropyl)carbamate (**2e**)

Following a general procedure, TFEO-ZnPc (5.4 mg, 0.0025 mmol, 1 mol %), Na ascorbate (17.3 mg, 0.0875 mmol, 0.35 equiv), alkene **1e** (39.3 mg, 0.25 mmol, 1.0 equiv) and C_8_F_17_I (99.0 μL, 0.375 mmol, 1.5 equiv) were used in MeCN (1.0 mL) and MeOH (0.75 mL) at room temperature for 1 h. The crude product was purified by column chromatography on silica gel (hexane/EtOAc = 8:2) to give perfluoroalkylated product **2e** (163.6 mg, 83% yield) as a white solid.

The ^1^H-NMR, ^19^F-NMR spectrum matched that reported in [19].

MS (ESI, *m*/*z*) 726 [(M + Na)^+^]; ^1^H-NMR (CDCl_3_, 300 MHz): δ 5.09–4.99 (m, 1H), 4.43–4.35 (m, 1H), 3.58–3.50 (m, 2H), 2.93–4.73 (m, 2H), 1.45 (s, 9H); ^19^F-NMR (CDCl_3_, 282 MHz): δ −81.2 (t, *J* = 8.7 Hz, 3F), −112.1–−114.7 (m, 2F), −121.9 (br s, 2F), −122.2 (br s, 4F), −123.1 (br s, 2F), −123.9 (br s, 2F), −126.5 (br s, 2F).

#### 3.1.9. (3-Iodo-4-perfluorooctylbutyl)benzene (**2f**)

Following a general procedure, TFEO-ZnPc (5.4 mg, 0.0025 mmol, 1 mol %), Na ascorbate (17.3 mg, 0.0875 mmol, 0.35 equiv), alkene **1f** (33.0 mg, 0.25 mmol, 1.0 equiv) and C_8_F_17_I (99.0 μL, 0.375 mmol, 1.5 equiv) were used in MeCN (1.0 mL) and MeOH (0.75 mL) at room temperature for 1 h. The crude product was purified by column chromatography on silica gel (hexane) to give perfluoroalkylated product **2f** (145.4 mg, 86% yield) as a white solid.

The ^1^H-NMR matched that reported in [52].

HRMS (EI) calcd. for C_18_H_12_F_17_I (M)^+^: 677.9712 found 677.9713; ^1^H-NMR (300 MHz, CDCl_3_): δ 7.31 (d, *J* = 6.0 Hz, 2H), 7.26–7.20 (m, 3H), 4.31–4.22 (m, 1H), 2.93–2.70 (m, 4H), 2.16–2.08 (m, 2H). ^19^F-NMR (282 MHz, CDCl_3_): δ −81.3 (t, *J* = 8.5 Hz, 3F), −111.3–−112.3 (m, 1F), −114.6–−115.6 (m, 1F), −122.1 (br s, 2F), −122.4 (br s, 4F), −123.2 (br s, 2F), −124.1 (br s, 2F), −126.6 (br s, 2F).

#### 3.1.10. 3-Iodo-4-perfluorooctyldobut-3-en-1-ol (**2g**)

Following a general procedure, TFEO-ZnPc (5.4 mg, 0.0025 mmol, 1 mol %), Na ascorbate (17.3 mg, 0.0875 mmol, 0.35 equiv), alkyne **1g** (17.5 mg, 0.25 mmol, 1.0 equiv) and C_8_F_17_I (99.0 μL, 0.375 mmol, 1.5 equiv) were used in MeCN (1.0 mL) and MeOH (0.75 mL) at room temperature for 1 h. The crude product was purified by column chromatography on silica gel (hexane/EtOAc = 8:2) to give perfluoroalkylated product **2g** (128.6 mg, 83% yield) as a white solid.

The ^1^H-NMR, ^19^F-NMR spectrum matched that reported in [19].

HRMS (EI) calcd. for C_18_H_12_F_17_I (M)^+^: 677.9712 found 677.9711; Data for major isomer of compound (**2g**); ^1^H-NMR (CDCl_3_, 300 MHz): δ 6.49 (t, *J* = 13.4 Hz, 1H), 3.89–3.85 (m, 2H), 2.97–2.92 (m, 2H), 1.70 (s, 1H); ^19^F-NMR (CDCl_3_, 282 MHz): δ −81.2 (t, *J* = 9.4 Hz, 3F), −105.3–−105.5 (m, 2F), −121.8 (br s, 2F), −122.3 (br s, 4F), −123.1 (br s, 2F), −123.4 (br s, 2F), −126.5 (br s, 2F). Data for minor isomer of compound (**2g**); ^1^H-NMR (CDCl_3_, 300 MHz): δ 6.41 (t, *J* = 12.1 Hz, 1H), 3.88–3.84 (m, 2H), 2.95–2.91 (m, 2H), 1.61 (s, 1H); ^19^F-NMR (CDCl_3_, 282 MHz): δ −81.2 (t, *J* = 10.0 Hz, 3F), −109.1–−109.2 (m, 2F), −121.8 (br s, 2F), −122.3 (br s, 4F), −123.2 (br s, 4F), −126.5 (br s, 2F).

#### 3.1.11. 2-Iodo-1-perfluorooctyloctene (**2h**)

Following a general procedure, TFEO-ZnPc (5.4 mg, 0.0025 mmol, 1 mol %), Na ascorbate (17.3 mg, 0.0875 mmol, 0.35 equiv), alkene **1h** (28.0 mg, 0.25 mmol, 1.0 equiv) and C_8_F_17_I (99.0 μL, 0.375 mmol, 1.5 equiv) were used in MeCN (1.0 mL) and MeOH (0.75 mL) at room temperature for 1 h. The crude product was purified by column chromatography on silica gel (hexane) to give perfluoroalkylated product **2h** (160.2 mg, 97% yield) as colorless oil.

HRMS (EI) calcd. for C_16_H_16_F_17_ [(M − I)^+^]: 531.0981 found 531.0997; ^1^H-NMR (CDCl_3_, 300 MHz): δ 4.38–4.30 (m, 1H), 3.03–2.68 (m, 2H), 1.87–1.75 (m, 2H), 1.55–1.52 (m, 8H), 0.91–0.90 (m, 3H); ^19^F-NMR (CDCl_3_, 282 MHz): δ −81.3 (t, *J* = 9.4 Hz, 3F), −111.8–−112.7 (m, 1F), −114.7–−115.7 (m, 1F), −122.1 (br s, 2F), −122.3 (br s, 4F), −123.2 (br s, 2F), −124.1 (br s, 2F), −126.7 (br s, 2F).; ^13^C NMR (CDCl_3_, 125 MHz): δ = 106.4–120.8 (m, C_8_F_17_), 41.7 (t, *J* = 20.6 Hz), 40.3 (apparent doublet, *J* = 1.3 Hz), 31.4, 29.5, 28.2, 22.5, 20.9, 14.0 (apparent doublet, *J* = 5.0 Hz); IR (NaCl) 2932, 2860, 1468, 1434, 1368, 1206, 1151, 705, 657, 559 cm^−1^.

#### 3.1.12. 5-Iodo-6-perfluorooctylhexane-2-one (**2i**)

Following a general procedure, TFEO-ZnPc (5.4 mg, 0.0025 mmol, 1 mol %), Na ascorbate (17.3 mg, 0.0875 mmol, 0.35 equiv), alkene **1i** (24.5 mg, 0.25 mmol, 1.0 equiv) and C_8_F_17_I (99.0 μL, 0.375 mmol, 1.5 equiv) were used in MeCN (1.0 mL) and MeOH (0.75 mL) at room temperature for 1 h. The crude product was purified by column chromatography on silica gel (hexane/EtOAc = 9:1) to give perfluoroalkylated product **2i** (137.6 mg, 85% yield) as a white solid.

m.p. = 42.4–43.3 °C; HRMS (EI) calcd. for C_14_H_10_F_17_O [(M − I)^+^]: 517.0460 found 517.0447; ^1^H-NMR (CDCl_3_, 300 MHz): δ 4.40–4.32 (m, 1H), 3.01–2.63 (m, 4H), 2.20–2.00 (m, 5H); ^19^F-NMR (CDCl_3_, 282 MHz): δ −81.2 (t, *J* = 8.5 Hz, 3F), −111.6–−112.6 (m, 1F), −114.4–−115.4 (m, 1F), −122.0 (br s, 2F), −122.3 (br s, 4F), −123.1 (br s, 2F), −124.0 (br s, 2F), −126.6 (br s, 2F).; ^13^C NMR (CDCl_3_, 125 MHz): δ = 206.5, 120.1–106.0 (m, C_8_F_17_), 43.7, 41.9 (t, *J* = 20.6 Hz), 34.0, 30.1, 19.8; IR (KBr) 2924, 2370, 1714, 1434, 1250, 1146, 1034, 705, 659 cm^−1^.

#### 3.1.13. 1-Iodo-2-perfluorooctylcyclohexane (**2j**)

Following a general procedure, TFEO-ZnPc (5.4 mg, 0.0025 mmol, 1 mol %), Na ascorbate (17.3 mg, 0.0875 mmol, 0.35 equiv), alkene **1j** (20.5 mg, 0.25 mmol, 1.0 equiv) and C_8_F_17_I (99.0 μL, 0.375 mmol, 1.5 equiv) were used in MeCN (1.0 mL) and MeOH (0.75 mL) at room temperature for 1 h. The crude product was purified by column chromatography on silica gel (hexane) to give perfluoroalkylated product **2j** (150.2 mg, 96% yield) as a white solid.

The ^1^H-NMR spectrum matched that reported in [53].

HRMS (EI) calcd. for C_16_H_16_F_17_ [(M − I)^+^]: 501.0511 found 501.0511; Data for major isomer of compound (**2j**); ^1^H-NMR (CDCl_3_, 300 MHz): δ 4.99–4.95 (m, 1H), 2.75–2.63 (m, 1H), 2.19–1.59 (m, 8H); ^19^F-NMR (CDCl_3_, 282 MHz): δ −81.2 (t, *J* = 8.9 Hz, 3F), −108.6–−109.6 (m, 1F), −110.3–−111.3 (m, 1F), −121.0–−121.3 (br s, 2F), −122.0–−122.3 (m, 6F), −123.1 (br s, 2F), −126.5 (br s, 2F). Data for minor isomer of compound (**2j**); ^1^H-NMR (CDCl_3_, 300 MHz): δ 4.74–4.70 (m, 1H), 2.24–2.20 (m, 1H), 2.00–1.37 (m, 8H); ^19^F-NMR (CDCl_3_, 282 MHz): δ −81.2 (t, *J* = 8.9 Hz, 3F), −118.0 (br s, 2F), −120.3–−122.2 (m, 8F), −123.1 (br s, 2F), −126.5 (br s, 2F).

#### 3.1.14. 2-Iodo-3-perfluorooctylnorbornane (**2k**) 

Following a general procedure, TFEO-ZnPc (5.4 mg, 0.0025 mmol, 1 mol %), Na ascorbate (17.3 mg, 0.0875 mmol, 0.35 equiv), alkene **1k** (23.5 mg, 0.25 mmol, 1.0 equiv) and C_8_F_17_I (99.0 μL, 0.375 mmol, 1.5 equiv) were used in MeCN (1.0 mL) and MeOH (0.75 mL) at room temperature for 1 h. The crude product was purified by column chromatography on silica gel (hexane) to give perfluoroalkylated product **2k** (69.8 mg, 44% yield) as a white solid.

The ^1^H-NMR spectrum matched that reported in [53].

HRMS (EI) calcd. for C_15_H_10_F_17_ [(M − I)^+^]: 513.0511 found 513.0497; ^1^H-NMR (CDCl_3_, 300 MHz): δ 4.33–4.31 (m, 1H), 2.50–2.31 (m, 3H), 1.93–1.30 (m, 6H) ; ^19^F-NMR (CDCl_3_, 282 MHz): δ −81.3 (t, *J* = 9.3 Hz, 3F), −115.6–116.6 (m, 1F), −119.0–−120.0 (m, 1F), −121.3 (br s, 2F), −122.2–−122.5 (m, 6F), −123.2 (br s, 2F), −126.6 (br s, 2F).

#### 3.1.15. 3-Iodo-4-(perfluorooctyl)butanenitrile (**2l**) 

Following a general procedure, TFEO-ZnPc (5.4 mg, 0.0025 mmol, 1 mol %), Na ascorbate (17.3 mg, 0.0875 mmol, 0.35 equiv), alkene **1l** (17.8 mg, 0.25 mmol, 1.0 equiv) and C_8_F_17_I (99.0 μL, 0.375 mmol, 1.5 equiv) were used in MeCN (1.0 mL) and MeOH (0.75 mL) at room temperature for 1 h. The crude product was purified by column chromatography on silica gel (hexane/EtOAc = 9:1) to give perfluoroalkylated product **2l** (33.0 mg, 22% yield) as a yellow solid.

m.p. = 81.7–82.7 °C; HRMS (EI) calcd. for C_12_H_5_F_17_NI [(M^+^)]: 612.9195 found 612.9209; ^1^H-NMR (CDCl_3_, 300 MHz): δ 4.49–4.41(m, 1H), 3.34–3.16 (m, 2H), 3.03–2.88 (m, 2H); ^19^F-NMR (CDCl_3_, 282 MHz): δ −81.2 (t, *J* = 9.4 Hz, 3F), −111.8–−112.7 (m, 1F), −114.9–−115.9 (m, 1F), −122.1 (br s, 2F), −122.4 (br s, 4F), −123.2 (br s, 2F), −123.9 (br s, 2F), −126.6 (br s, 2F); ^13^C NMR (CDCl_3_, 125 MHz): δ =120.0– 108.3 (m, C_8_F_17_), 116.4, 40.5 (t, *J* = 21.3 Hz), 30.3 (apparent doublet, *J* = 3.8 Hz), 5.34 (apparent doublet, *J* = 3.8 Hz); IR (KBr) 2958, 2920, 2366, 2258, 1371, 1203, 1149, 1117, 972, 657 cm^−1^.

## 4. Conclusions

In summary, we disclose the first photo-induced radical perfluoroalkylation of alkenes and alkyne induced by trifluoroethoxy-coated zinc phthalocyanine as a catalyst. From the view of the ease of availability, lower cost, and the substantiality of phthalocyanines, this study will be a monumental work of phthalocyanines as photocatalysts. Further studies to reveal the new potential of phthalocyanines are under investigation by our group [54].

## Figures and Tables

**Table 1:**
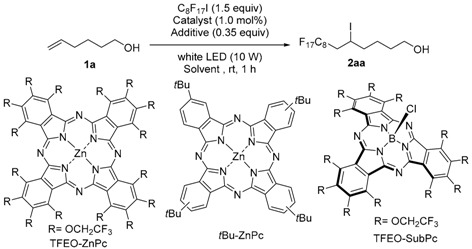
Perfluorooctylation reaction of 1-hexenol with TFEO-ZnPc under visible light irradiation. ^a^

Entry	Catalyst (1 mol %)	Additive (0.35 equiv)	Solvent	Yield (%) ^b^
1	TFEO-ZnPc	Na ascorbate	MeCN/MeOH	88
2 ^c^	TFEO-ZnPc	Na ascorbate	MeCN/MeOH	<5
3	-	Na ascorbate	MeCN/MeOH	<5
4	TFEO-ZnPc	-	MeCN/MeOH	<5
5	*t*Bu-ZnPc	Na ascorbate	MeCN/MeOH	45
6	TFEO-SubPc	Na ascorbate	MeCN/MeOH	77
7	TFEO-ZnPc	Ascorbic acid	MeCN/MeOH	33
8	TFEO-ZnPc	Hantzsch ester	MeCN/MeOH	24
9 ^d,e^	TFEO-ZnPc	Na ascorbate	MeCN	<5
10 ^d^	TFEO-ZnPc	Na ascorbate	MeOH	62
11 ^f^	TFEO-ZnPc	Na ascorbate	DMSO	7
12 ^g^	TFEO-ZnPc	Na ascorbate	MeCN/MeOH	93

^a^ The reaction of 1-hexenol (**1a** 0.25 mmol) with *n*C_8_F_17_I (0.375 mmol) was carried out in the presence of TFEO-ZnPc (0.0025 mmol) and Na ascorbate (0.0875 mmol) in MeCN (2.0 mL) and MeOH (1.5 mL) at room temperature under irradiation with white LED (10 W); ^b^ Yields were calculated by ^19^F-NMR of crude product using PhCF_3_ as an internal standard; ^c^ Reaction was carried out in the dark; ^d^ Reaction time was 24 h; ^e^ Tetrabutylammonium bromide (TBAB, 10 mol %) was added; ^f^ Reaction was carried out for 5 h without Na ascorbate; ^g^ Reaction was carried out in MeCN (1.0 mL) and MeOH (0.75 mL). TFEO-ZnPc, trifluoroethoxy-coated zinc phthalocyanine; *t*BuZnPc, *t*Bu-functionalized zinc phthalocyanine; TFEO-SubPc , trifluoroethoxy-coated subphthalocyanine.
